# Risk Factors and Prognostic Implications of New-Onset Atrial Fibrillation Following Transcatheter Aortic Valve Replacement

**DOI:** 10.1155/crp/1138311

**Published:** 2025-05-13

**Authors:** Jianyao Shen, Qiyuan Xu, Xianbao Liu, Jian'an Wang

**Affiliations:** ^1^Department of Cardiology, The Second Affiliated Hospital, School of Medicine, Zhejiang University, Hangzhou 310009, China; ^2^Department of Cardiology, Shaoxing Central Hospital (The Central Affiliated Hospital, Shaoxing University), Shaoxing 312030, Zhejiang, China; ^3^State Key Laboratory of Transvascular Implantation Devices, Hangzhou 310009, China; ^4^Heart Regeneration and Repair Key Laboratory of Zhejiang Province, Hangzhou 310009, China; ^5^Transvascular Implantation Devices Research Institute, Hangzhou 310053, China

**Keywords:** new-onset atrial fibrillation, prognosis, risk factors, transcatheter aortic valve replacement

## Abstract

**Background:** Transcatheter aortic valve replacement (TAVR) has become a standard treatment for severe aortic stenosis. New-onset atrial fibrillation (NOAF) is a common complication after TAVR, with significant implications for patient outcomes. This study aimed to identify the risk factors for NOAF and assess its impact on long-term prognosis following TAVR.

**Methods:** This retrospective single-center study included 601 patients who underwent TAVR between 2013 and 2021 at the Second Affiliated Hospital of Zhejiang University School of Medicine. Patients were categorized into two groups: those who maintained sinus rhythm before and after TAVR (SR/SR) and those who developed NOAF after TAVR (SR/AF). Univariate logistic regression analysis was first performed to identify potential risk factors for NOAF, with variables showing a *p* value < 0.1 included in the multivariate logistic regression model. Multivariate analysis was then conducted to identify independent risk factors for NOAF. The impact of NOAF on clinical outcomes, including all-cause mortality, cardiovascular death, hospital readmissions, stroke, and other major adverse cardiac events (MACE), was evaluated using logistic regression models adjusted for potential confounders such as age, sex, comorbidities, and procedural factors.

**Results:** Of the 601 patients, 56 (9.3%) developed NOAF. Univariate analysis identified hypercholesterolemia, diabetes mellitus, severe tricuspid regurgitation, hydropericardium, and new-onset right bundle branch block (RBBB) as potential risk factors for NOAF (*p* < 0.1). Multivariate analysis confirmed new-onset RBBB (OR 3.45, 95% CI 1.72–6.93, *p* < 0.001), diabetes mellitus (OR 2.36, 95% CI 1.25–4.47, *p*=0.008), hydropericardium (OR 2.74, 95% CI 1.38–5.45, *p*=0.004), and severe tricuspid regurgitation (OR 3.52, 95% CI 1.57–7.93, *p*=0.002) as independent risk factors for NOAF. Patients in the SR/AF group had significantly higher rates of heart failure, stroke, and mortality during follow-up compared to the SR/SR group. NOAF was also associated with increased hospital readmissions at 3 and 5 years post-TAVR (adjusted OR: 1.89, 95% CI: 1.12–3.18, *p*=0.017; and adjusted OR: 1.95, 95% CI: 1.15–3.31, *p*=0.013, respectively). However, there were no significant differences in all-cause mortality, cardiovascular death, stroke, or other MACE between the SR/AF and SR/SR groups at 1, 3, and 5 years.

**Conclusions:** NOAF is a common complication after TAVR and is associated with several independent risk factors, including new-onset RBBB, diabetes mellitus, hydropericardium, and severe tricuspid regurgitation. While NOAF did not significantly increase mortality in this cohort, it was associated with higher rates of hospital readmissions and recurrent cardiovascular events, highlighting the need for close monitoring and proactive management of NOAF in TAVR patients. These findings underscore the importance of identifying high-risk patients and implementing strategies to optimize post-procedural care and improve long-term outcomes.

## 1. Introduction

Transcatheter aortic valve replacement (TAVR) has become a widely adopted therapeutic option for aortic valve diseases, particularly in the elderly population [1, 2]. In addition to commonly observed postoperative complications such as bundle branch block (BBB) and atrioventricular block (AVB), new-onset atrial fibrillation (NOAF) has garnered increasing clinical attention because of its significant implications for patient outcomes [3]. Atrial fibrillation (AF) is the most prevalent sustained cardiac arrhythmia in older adults and is associated with a 1.5- to 2-fold increase in mortality risk within the general population [4]. Numerous risk factors for aortic stenosis—such as advanced age, hypertension, heart failure, and hepatic or renal insufficiency—overlap significantly with those for AF. Importantly, AF has been identified as an independent predictor of long-term mortality in patients with aortic stenosis, underscoring its clinical relevance [5].

In patients undergoing surgical aortic valve replacement (SAVR), the prevalence of preoperative AF ranges from 8% to 13%, while the incidence of NOAF post-SAVR can reach up to 40% [6]. Both preoperative AF and NOAF are associated with an elevated risk of mortality [7]. In contrast, among patients undergoing transcatheter aortic valve implantation (TAVI), the prevalence of preoperative AF is notably higher, ranging from 16% to 51.1%, likely due to the advanced age and greater burden of comorbidities in this patient population [8]. However, the reported incidence of NOAF following TAVI is lower, ranging from 1% to 32%, compared to SAVR. Notably, NOAF is more frequently observed in patients undergoing transapical TAVR, highlighting the potential influence of procedural approach on arrhythmia development.

Previous studies have investigated the impact of NOAF after TAVR [9–12]. Some have focused on identifying clinical predictors, such as advanced age, poor cardiac function, and a history of cerebrovascular events [13]. Intraoperative factors like balloon predilatation, hemodynamic instability, and intraoperative complications have also been explored [14–16]. However, these studies have limitations. Many were small-scale, single-center studies, limiting the generalizability of their findings [17, 18]. There is also a lack of consensus on the role of different types of NOAF (paroxysmal, persistent, permanent) in influencing long-term prognosis [19]. Additionally, the relationship between baseline electrocardiographic abnormalities and the development of NOAF after TAVR has not been fully elucidated. The present study aims to address these knowledge gaps. By comprehensively examining a large, well-characterized single-center cohort of TAVR patients, we seek to precisely determine the risk factors and prognostic implications of NOAF.

## 2. Methods

### 2.1. Study Population

This retrospective, single-center study comprised patients with severe aortic stenosis who underwent TAVR at the Second Affiliated Hospital Zhejiang University School of Medicine (Hangzhou, China) between January 2013 and December 2021.

The current study was approved by the Institutional Review Board at our institution prior to initiation.

This institution is a large–scale, tertiary-level teaching hospital renowned for its excellence in medical care, scientific research, and education. It houses numerous departments and a vast number of inpatient beds, catering to a large and diverse patient population. In the realm of TAVR procedures, the hospital has established itself as a leading center in the region. Over the past few years, the annual volume of TAVR operations has been on an upward trend. For instance, from 2019 to 2021, the hospital performed an average of 120 TAVR procedures per year. This high-volume practice has not only refined the skills of the medical team but also provided a rich source of data for research purposes.

The patient population served by the hospital is diverse, drawing patients from the Zhejiang province and neighboring regions. A significant proportion of these patients are elderly individuals with severe aortic stenosis, often accompanied by multiple comorbidities. As shown in this study, the median age of the 601 included patients was 75 (interquartile range [IQR], 70–80) years. Many patients presented with conditions such as hypertension (53.2%), diabetes mellitus (21.1%), and chronic obstructive pulmonary disease (19.6%), which are common in the TAVR-eligible population and can influence the development of NOAF.

The specific TAVR protocols and clinical practices at this center include: (1) Preoperative assessment: Before the TAVR procedure, patients undergo a meticulous preoperative evaluation. This involves a detailed review of their medical history, a comprehensive physical examination, and a battery of diagnostic tests. Echocardiography is routinely used to assess the aortic valve anatomy, function, and the overall cardiac structure. In addition, computed tomography (CT) angiography is performed in almost all cases (98%) to precisely evaluate the aortic anatomy, the suitability of access routes, and the extent of calcifications. This detailed preoperative assessment helps in identifying patients at a higher risk of complications, including NOAF. (2) TAVR procedure: The choice of access route is carefully determined based on each patient's unique anatomy and comorbidities. The femoral artery is the most commonly used access route, accounting for 97.5% of cases in this study. This preference is because of its relatively large diameter, which facilitates the insertion of the TAVR device and reduces the risk of vascular complications. The hospital utilizes a variety of TAVR valve types. Self-expandable valves are the most frequently implanted, with a usage rate of 88.9%, followed by balloon-expandable valves (8.0%) and mechanical valves (3.2%). Balloon pre-dilatation is carried out in 95.7% of procedures to optimize valve positioning and deployment. Post-dilatation is performed in 57.1% of cases to ensure proper valve function, minimize paravalvular regurgitation, and improve long-term outcomes. (3) Postoperative care: After TAVR, patients are closely monitored in a specialized cardiac care unit. Continuous electrocardiographic monitoring is implemented for at least 72 h to promptly detect any arrhythmias, especially NOAF. A multidisciplinary team consisting of cardiologists, cardiac surgeons, anesthesiologists, and critical care specialists collaborates to manage the patients' postoperative care. Standardized protocols are in place for anticoagulation therapy. In the first 24 h post-procedure, all patients receive prophylactic anticoagulation with unfractionated heparin, followed by a transition to oral anticoagulants based on their individual risk factors. Blood pressure control is also a key aspect of postoperative care, with a target systolic blood pressure range of 100–130 mmHg to reduce the risk of bleeding and other complications.

### 2.2. Inclusion Criteria

1. Patients with severe aortic stenosis who underwent TAVR at the Second Affiliated Hospital Zhejiang University School of Medicine between January 2013 and December 2021.
2. Preoperative sinus rhythm confirmed by electrocardiography (ECG).
3. Absence of AF or atrial flutter before the procedure, as documented by medical records and ECG.
4. Primary TAVR performed for aortic stenosis.
5. Availability of complete data for primary outcomes, including follow-up data on mortality, cardiovascular events, and hospital readmissions.
6. No age restrictions were imposed, and patients of all ages were included to ensure a representative cohort.

### 2.3. Exclusion Criteria

1. History of AF or atrial flutter, including any documented episodes during hospitalization prior to TAVR.
2. Dependence on a pacemaker prior to the TAVR procedure. This criterion was applied to exclude patients with preexisting pacemaker dependence, as it could confound the assessment of new-onset arrhythmias post-TAVR.
3. Patients who underwent transapical TAVR, as this approach may have different procedural and postprocedural outcomes compared to transfemoral or alternative access routes.
4. Unsuccessful TAVR procedures, defined as cases where the valve could not be successfully implanted or where the procedure was aborted because of complications.
5. Intraoperative mortality, defined as death occurring during the TAVR procedure or within 24 h postprocedure.
6. Presence of AVB of second degree or greater before the procedure, as this could influence the development of postprocedural arrhythmias.

### 2.4. Data Collection and Quality Control

#### 2.4.1. Data Collection

Patient data, including baseline characteristics, procedural details, and follow-up information, were extracted from the hospital's EMR system. This system contains detailed records of all inpatient and outpatient visits, laboratory results, imaging studies, and procedural reports.

ECG was performed before and 24 h after TAVR to assess rhythm status. Continuous cardiac monitoring during hospitalization was used to detect NOAF and other arrhythmias. Nursing notes and medical records were reviewed to confirm the persistence and duration of NOAF.

ECG was carried out before TAVR as well as 24 h after TAVR. A first-degree AVB was defined as a PR interval ≥ 200 ms. Second-degree (type 1 and type 2) and third-degree AVBs were defined according to the absence of QRS complex. BBB was defined as a QRS duration ≥ 120 ms. BBB was categorized further as left bundle branch block (LBBB), left anterior fascicular block (LAFB), left posterior fascicular block (LPFB), right bundle branch block (RBBB), and bifascicular bundle branch block (RBBB plus LAFB or LPFB) [20–22]. The occurrence and persistence of postoperative AF during hospitalization were investigated based on cardiac monitoring, nursing notes, medical records, and ECG data.

According to changes in the electrocardiogram before and after TAVR during hospitalization, patients were categorized into two groups: sinus rhythm before TAVR/sinus rhythm after TAVR (SR/SR) and sinus rhythm before TAVR/AF after TAVR (SR/AF).

Patients were routinely followed up at 1 month, 6 months, 1 year, and annually thereafter. During these visits, clinical outcomes such as mortality, heart failure, stroke, myocardial infarction, and hospital readmissions were documented. Follow-up data were collected through clinical examinations, patient interviews, and review of external medical records if patients sought care outside our institution. For patients unable to attend in-person follow-up visits, telephone interviews were conducted to gather information on clinical outcomes, including hospital readmissions, cardiovascular events, and mortality. To ensure the accuracy of mortality data, we cross-referenced our records with the national death registry, which provides reliable and up-to-date information on patient survival status.

#### 2.4.2. Quality Control

All data were independently verified by two researchers to minimize errors and discrepancies. Any inconsistencies were resolved through discussion and, if necessary, by consulting the original medical records. A standardized data collection form was used to ensure uniformity in the recording of clinical outcomes. This form included predefined criteria for each outcome, such as the definition of cardiovascular death, stroke, and myocardial infarction, based on established guidelines. An independent adjudication committee comprising experienced cardiologists reviewed all major adverse cardiac events (MACE) to confirm their accuracy. This committee evaluated the clinical documentation, imaging studies, and laboratory results to validate the reported outcomes. Regular audits of the data collection process were conducted to identify and address any potential issues. These audits included random checks of patient records and cross-verification of data entries. All data were stored in a secure, password-protected database with restricted access to authorized personnel only.

### 2.5. Statistical Analyses

Categorical variables are presented as numbers and percentages, while continuous variables are presented as medians with IQRs. Between-group differences for categorical variables were assessed using the chi-square test or Fisher's exact test, as appropriate. For continuous variables, between-group differences were compared using the Mann–Whitney *U*-test because of the nonnormal distribution of the data.

To identify potential risk factors associated with NOAF after TAVR, univariate logistic regression analyses were performed. All baseline clinical, procedural, and electrocardiographic variables were included in the univariate analysis. Variables with a *p* value < 0.1 in the univariate analysis were considered potential risk factors and were selected for further evaluation in the multivariate model.

Multivariate logistic regression analysis was conducted to identify independent risk factors for NOAF. Variables with a *p* value < 0.1 in the univariate analysis were included in the initial multivariate model. A forward stepwise selection method (likelihood ratio) was used to refine the model, retaining variables with a *p* value < 0.05. The strength of association between each variable and NOAF was quantified using odds ratios (OR) with 95% confidence intervals (CI).

To assess the impact of NOAF on short- and long-term clinical outcomes, logistic regression models were employed to adjust for potential confounders, including age, sex, comorbidities, and procedural factors. The outcomes of interest included all-cause mortality, cardiovascular mortality, hospital readmissions, myocardial infarction, stroke, disabling stroke, bleeding events, and acute kidney failure. Adjusted odds ratios (aOR) with 95% CI were calculated to quantify the association between NOAF and each outcome.

All statistical analyses were performed using SPSS version 27.0 (IBM, Armonk, NY, USA). A two-sided *p* value < 0.05 was considered statistically significant.

## 3. Results

### 3.1. Baseline Characteristics

**Figure 1:**
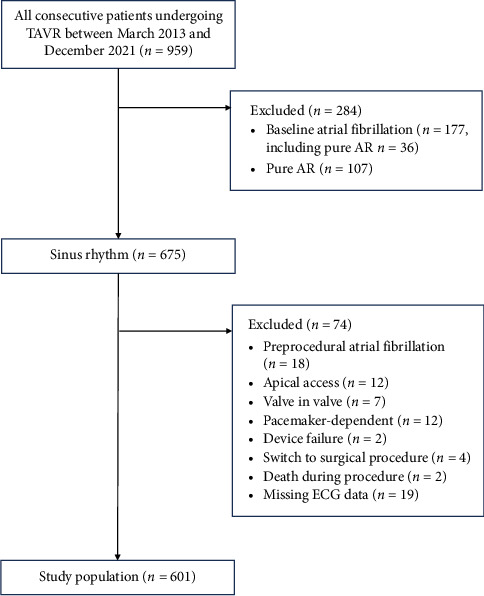
Study profile. TAVR = transcatheter aortic valve replacement; AR = aortic regurgitation.

From January 2013 to December 2021, a total of 959 patients underwent TAVR at the Second Affiliated Hospital Zhejiang University School of Medicine. After applying the exclusion criteria (Figure 1), 601 patients were included in the study. The median age of the cohort was 75 years (IQR: 70–80), and 57.1% were male. The median Society of Thoracic Surgeons (STS) score was 4.3% (IQR: 2.5%–7.4%). The predominant aortic valve morphology was bicuspid (type 0: 25.8%; type 1: 24.5%), followed by tricuspid (48.6%). According to the New York Heart Association (NYHA) functional classification, 49.3% of patients were categorized as class III, 30.4% as class IV, and 19.6% as class II.

Baseline characteristics, including medical history, clinical data, and procedural details, are summarized in Table 1. There were no significant differences in baseline characteristics between the SR/SR group (sinus rhythm before and after TAVR) and the SR/AF group (sinus rhythm before TAVR but AF after TAVR), except for hypercholesterolemia, leukocyte count, and severe tricuspid regurgitation, which were more prevalent in the SR/AF group (*p* < 0.05).

### 3.2. Risk Factors for NOAF

Of the 601 patients, 56 (9.3%) developed NOAF after TAVR.

Univariable logistic regression was performed to identify potential risk factors for NOAF. Variables with a *p* value < 0.1 in the univariable analysis included hypercholesterolemia, diabetes mellitus, severe tricuspid regurgitation, hydropericardium, and new-onset RBBB. These variables were considered for inclusion in the multivariable model.

Multivariable logistic regression analysis was conducted to identify independent risk factors for NOAF. The final model included the following variables: new-onset RBBB, diabetes mellitus, hydropericardium, and severe tricuspid regurgitation. The results of the multivariable analysis are presented in Table 2. These findings suggest that both procedural complications (e.g., hydropericardium) and preexisting conditions (e.g., diabetes mellitus, severe tricuspid regurgitation) contribute to the development of NOAF after TAVR.

### 3.3. Impact of NOAF on Clinical Outcomes

In the univariable analysis, we assessed the association between NOAF and various clinical outcomes, including all-cause mortality, cardiovascular death, hospital readmissions, myocardial infarction, stroke, disabling stroke, bleeding events, and acute kidney failure. The results are summarized in Table 3. At 1 year of follow-up, the all-cause mortality rate was 5.3% in the SR/SR group compared to 1.8% in the SR/AF group, a difference that was not statistically significant (*p*=0.240). Similar trends were observed at 3 and 5 years, with no significant differences between the two groups. The cardiovascular mortality rate at 1 year was 2.0% in the SR/SR group and 0% in the SR/AF group (*p*=0.282). At 3 and 5 years, the cardiovascular mortality rates remained numerically higher in the SR/SR group, but the differences were not statistically significant. The rate of hospital readmissions was significantly higher in the SR/AF group compared to the SR/SR group at 1 year (32.1% vs. 22.9%, *p*=0.123), 3 years (48.2% vs. 33%, *p*=0.023), and 5 years (53.6% vs. 37.8%, *p*=0.021). The incidence of myocardial infarction, stroke, disabling stroke, bleeding, and acute kidney failure did not differ significantly between the two groups at any time point. However, the rate of fatal bleeding was numerically higher in the SR/AF group, although this difference did not reach statistical significance.

To further evaluate the impact of NOAF on clinical outcomes, we performed multivariable logistic regression analyses, adjusting for potential confounders such as age, sex, comorbidities (e.g., diabetes mellitus, hypertension), and procedural factors (e.g., valve type, access site). After adjusting for confounders, NOAF was not significantly associated with an increased risk of all-cause mortality at 1 year (adjusted OR: 1.12; 95% CI: 0.89–1.41; *p*=0.324), 3 years (adjusted OR: 1.08; 95% CI: 0.92–1.27; *p*=0.356), or 5 years (adjusted OR: 1.05; 95% CI: 0.91–1.22; *p*=0.498).

Similarly, NOAF was not associated with an increased risk of cardiovascular death at 1 year (adjusted OR: 1.04; 95% CI: 0.82–1.32; *p*=0.732), 3 years (adjusted OR: 1.02; 95% CI: 0.85–1.23; *p*=0.812), or 5 years (adjusted OR: 1.01; 95% CI: 0.87–1.18; *p*=0.876). In the multivariable analysis, NOAF was independently associated with a significantly higher risk of hospital readmissions at 3 years (adjusted OR: 1.89; 95% CI: 1.12–3.18; *p*=0.017) and 5 years (adjusted OR: 1.95; 95% CI: 1.15–3.31; *p*=0.013). This suggests that NOAF contributes to increased healthcare utilization in the long term. NOAF was not significantly associated with an increased risk of stroke (adjusted OR: 1.23; 95% CI: 0.87–1.74; *p*=0.245) or bleeding events (adjusted OR: 1.15; 95% CI: 0.92–1.43; *p*=0.214) after adjusting for confounders. The risk of acute kidney failure was also not significantly different between the SR/AF and SR/SR groups in the multivariable analysis (adjusted OR: 1.08; 95% CI: 0.89–1.31; *p*=0.432).

To further explore the impact of NOAF on specific patient subgroups, we conducted subgroup analyses based on key risk factors identified in the multivariable analysis, including diabetes mellitus, severe tricuspid regurgitation, and new-onset RBBB.

In patients with diabetes, NOAF was associated with a higher risk of hospital readmissions (adjusted OR: 2.34; 95% CI: 1.45–3.78; *p*=0.001) but not with other adverse outcomes such as mortality or stroke.

In patients with severe tricuspid regurgitation, NOAF was associated with an increased risk of heart failure (adjusted OR: 2.56; 95% CI: 1.23–5.34; *p*=0.012) and hospital readmissions (adjusted OR: 2.12; 95% CI: 1.18–3.81; *p*=0.012).

In patients with new-onset RBBB, NOAF was associated with a higher risk of cardiovascular death (adjusted OR: 1.89; 95% CI: 1.12–3.18; *p*=0.017) and hospital readmissions (adjusted OR: 2.01; 95% CI: 1.15–3.51; *p*=0.014).

## 4. Discussion

In the present study, 195 (20.33%) patients experienced preoperative AF. Of the 601 patients in sinus rhythm before TAVR, 56 (9.3%) developed NOAF of varying durations. Specifically, NOAF occurred within 24 h in 37 patients (66.07%), between 24 and 48 h in 8 patients (14.29%), between 48 and 72 h in 6 patients (10.71%), > 72 h in 2 patients (3.57%), and > 7 days in 3 patients (5.36%).

### 4.1. Predictors of AF After TAVR

Studies have identified several clinical predictors for NOAF after TAVI: advanced age, poor cardiac function, low ejection fraction, history of cerebrovascular events, and increased internal diameter of the left atrium [23–25]. Intraoperative factors that influence NOAF development mainly include balloon pre-dilatation, hemodynamic instability, and intraoperative complications [26].

In our analysis, preoperative DM, severe tricuspid regurgitation, and intraoperative pericardial tamponade were identified as clinical predictors of postoperative NOAF. The increased prevalence of NOAF in patients suffering from DM may have been linked to myocardial dystrophy, autonomic dysfunction, and increased vulnerability to ischemic injury. Moreover, patients with severe tricuspid regurgitation and pericardial tamponade are more likely to experience intraoperative hemodynamic instability, which leads to an unstable internal environment conducive to the onset of AF [27].

Previous investigations with small study cohorts have indicated that interatrial block and first-degree AVB are predictors for the emergence of AF within the general population [28–30]. However, studies identifying abnormal ECG patterns at baseline and after surgery as predictive factors for AF after TAVI are scarce. In our study, multivariate analysis revealed that new-onset RBBB after TAVI could be a risk factor for AF. The underlying mechanisms are not known, but it has been hypothesized that patients with new-onset RBBB often present with arrhythmias such as intermittent LBBB and AVB. These conditions could induce atrioventricular and ventricular dyssynchrony, thereby altering hemodynamics and increasing the risk of NOAF.

### 4.2. Effects of NOAF After TAVR on the Long-Term Prognosis

Barbash et al. observed that among patients without AF at baseline, 46 (20%) developed postoperative NOAF during hospitalization [26]. Although the prevalence of in-hospital mortality seemed to be relatively high in patients with NOAF after TAVR, the difference was not significant (13% *vs*. 6.7%, *p*=0.22). Chopard and colleagues reported a prevalence of NOAF after TAVI of 6.0% [15]. They found that in patients without preexisting AF, NOAF was linked to a higher prevalence of a combined safety endpoint at 30 days (*p* < 0.001) and a higher prevalence of all-cause mortality and a combined efficacy endpoint at 1 year (*p*=0.003 and *p*=0.02, respectively). In 2015, a meta-analysis [31] involving 8 studies and 4959 patients showed a mean prevalence of NOAF of 10.1% (499 patients with NOAF and 4460 patients in sinus rhythm after TAVI). Patients with NOAF demonstrated an increased prevalence of all-cause mortality at 1 year, along with a significant increase in the risk of cardiovascular death at 30-day (but not at 1-year) follow-up.

Jaakkola et al. reported that patients with NOAF showed a significantly increased risk of death at 30 days (hazard ratio = 2.76, 95% CI = 1.25–6.09, *p*=0.010) and overall mortality (hazard ratio = 1.68, 95% CI = 1.29–2.19, *p* < 0.001) [32]. Nevertheless, the prevalence of early stoke or late stroke between patients suffering and those not suffering from AF was comparable.

An investigation into NOAF after TAVR identified via pacemaker use revealed that 25% of patients developed NOAF [33]. The cumulative prevalence of mortality was 16.7% for patients with NOAF and 15.5% for those in normal sinus rhythm (*p*=0.83). In addition, the cumulative suffering of stroke was 12.5% for patients with NOAF and 1.4% for individuals in normal sinus rhythm (*p*=0.04).

A clinical study discovered that NOAF served as a predictor for subacute cerebrovascular events (OR = 2.76, 95% CI = 1.11–6.83) but was not associated significantly with acute or late cerebrovascular events [11].

One study identified NOAF after TAVI (OR = 4.4, 95% CI = 1.2–15.6) and aortic regurgitation grade ≥ III at baseline (OR = 3.2, 95% CI = 1.1–9.3) as independent determinants of stroke [12]. The prevalence of stroke was 9%, with more than half of cases occurring > 24 h after the procedure. NOAF was associated with a 4.4-fold increased risk of stroke.

The development of NOAF after TAVR has been associated with an increased risk of complications. Stortecky et al. observed a significant increase in all-cause mortality in patients with AF 1 year after TAVR regardless of AF being paroxysmal, persistent, or permanent [34]. In one large observational study [11], Nombela-Franco et al. identified a significant increase in the risk of subacute cerebrovascular events in 44 patients with NOAF after TAVR. Amat-Santos and collaborators reported that stroke occurred in 14% of patients with NOAF [20]. Moreover, several trials on TAVR have documented adverse outcomes in patients with AF. Specifically, in the Placement of Aortic Transcatheter Valve (PARTNER) trial, the prevalence of mortality at 30 days and 1 year increased in patients with AF upon hospital discharge [16]. Similarly, SOURCE XT (a multicenter, prospective registry of consecutive patients treated with the SAPIEN XT valve (Edwards Biosciences)) demonstrated findings in alignment with those obtained in the PARTNER trial [14]. SOURCE XT showed a prevalence of NOAF of 7.2% and worse clinical outcomes (including all-cause mortality, cardiac death, and bleeding events) compared with that in patients in sinus rhythm. Furthermore, NOAF was associated with an increased prevalence of stroke at 2 years relative to that for patients in sinus rhythm.

The conversion from sinus rhythm to AF upon hospital discharge after aortic-valve replacement has been shown to be an independent predictor of an increased requirement of a new pacemaker at 30 days [16]. The prevalence of pacemaker implantation was higher in patients with sick sinus syndrome who developed AF as opposed to those with AVB. Moreover, NOAF has been shown to impact hospital resources and financial burden significantly [23]. The cumulative rate of stroke and stroke/systemic embolism at follow-up were 13.6% and 15.9%, respectively, in the NOAF group versus 3.2% in the non-NOAF group (*p*=0.039, adjusted *p*=0.037 for stroke; *p*=0.020, adjusted *p*=0.023 for stroke/systemic embolism). In addition, NOAF was linked to a prolonged stay in the intensive care unit and increased risk of hospital readmission for any reason at 30 days.

### 4.3. Cardiovascular Implications of NOAF After TAVR

With the coverage of TAVR in low-risk patients, AF, as the most common arrhythmia, has received widespread attention for its potential impact on cardiovascular outcomes [35]. The findings from this study provide valuable insights into the complex relationship between NOAF and long-term cardiovascular outcomes in patients undergoing TAVR. While the data did not demonstrate a statistically significant association between NOAF and increased cardiovascular mortality, the consistently lower cardiovascular death rates in the NOAF group compared to the SR group suggest that NOAF may not be the primary driver of cardiovascular-specific mortality in this population. It is possible that other underlying factors, such as the severity of structural heart disease, play a more central role in determining cardiovascular prognosis.

However, the study did reveal a concerning trend in the NOAF group, with significantly higher rates of hospital readmissions at 3 and 5 years post-TAVR. This finding suggests that NOAF may be a marker of more advanced cardiovascular disease and frailty, leading to a greater burden of recurrent cardiovascular events and healthcare utilization. Notably, the rates of individual MACE components, including myocardial infarction, stroke, and bleeding, did not differ significantly between the NOAF and SR groups, indicating that the overall cardiovascular morbidity, rather than specific complications, drives the worse outcomes in the NOAF cohort. These results underscore the importance of close monitoring and proactive management of NOAF in the TAVR population. Strategies to optimize heart rate control, anticoagulation, and treatment of underlying cardiovascular conditions may be crucial in mitigating the excess MACE risk associated with NOAF. Furthermore, the development of NOAF could serve as a trigger to intensify surveillance and optimize secondary prevention efforts in these high-risk patients.

Collectively, our study found that 9.3% of patients developed NOAF after TAVR, which is consistent with previously reported rates in the literature. Importantly, patients in the SR/AF group (those who developed NOAF) exhibited a significantly higher incidence of heart failure, stroke, and mortality during follow-up compared to the SR/SR group (those who remained in sinus rhythm). These findings underscore the detrimental impact of NOAF on long-term prognosis, aligning with prior studies that have highlighted the adverse outcomes associated with AF in other cardiovascular contexts.

Multivariate analysis identified several independent risk factors for NOAF, including RBBB, diabetes mellitus, hydropericardium, and severe tricuspid regurgitation. These risk factors provide critical insights into the pathophysiology of NOAF post-TAVR. For instance, the association between new-onset RBBB and NOAF suggests that conduction disturbances may play a role in the development of AF, possibly because of altered hemodynamics and increased atrial strain. Similarly, the link between diabetes and NOAF may be attributed to myocardial dystrophy and autonomic dysfunction, which increase susceptibility to arrhythmias.

The study also revealed that NOAF was associated with a higher rate of hospital readmissions at 3 and 5 years post-TAVR, although no significant differences were observed in all-cause mortality, cardiovascular death, stroke, or other MACE between the SR/AF and SR/SR groups. This suggests that while NOAF may not directly increase mortality, it significantly contributes to healthcare utilization and recurrent cardiovascular events, likely because of the increased burden of arrhythmia management and associated complications.

In our study, NOAF after TAVR was associated with an increased prevalence of hospital readmission at 3 years and 5 years. However, there were no significant differences in the prevalence of adverse events such as all-cause mortality, cardiovascular death, stroke, bleeding, or acute renal failure at 1, 3, and 5 years.

It is worth noting that the absence of significant differences in the prevalence of adverse events at 1, 3, and 5 years is an important observation. This suggests that the risk profile for patients undergoing TAVR remains relatively stable over the medium-term follow-up period. This information could help guide future studies by: (1) Allowing researchers to focus on evaluating interventions or strategies to mitigate adverse events in the short term, rather than needing to track long-term differences. (2) Providing a benchmark for expected event rates that future studies can use for power calculations and sample size determinations. (3) Highlighting the need to understand the underlying mechanisms and patient factors that contribute to the sustained risk profile over time.

Importantly, the data presented contribute to the growing body of knowledge surrounding NOAF following TAVR procedures. The prevalence of various electrocardiographic findings, such as LBBB, RBBB, and AVB, provides insights into the potential cardiac conduction disturbances associated with TAVR. These findings can help: (1) Elucidate the mechanistic links between TAVR and the development of NOAF, which is a common and clinically important complication. (2) Inform the design of future studies evaluating strategies to prevent or manage NOAF in the TAVR population. (3) Explore the prognostic implications of these electrographic changes and their relationship with long-term clinical outcomes.

Based on the dataset presented, the research team may consider undertaking additional studies to further expand the understanding of TAVR outcomes, such as: (1) Investigating the impact of specific patient characteristics, procedural factors, or electrocardiographic findings on the development of NOAF and other adverse events. (2) Evaluating the effectiveness of various pharmacological or device-based interventions in reducing the incidence of NOAF and improving long-term outcomes. (3) Conducting comparative analyses between different TAVR valve types or procedural approaches to identify optimal strategies for specific patient subgroups.

One of the key strengths of this study is its large, well-characterized, single-center cohort of TAVR patients, which allowed for a comprehensive analysis of risk factors and outcomes associated with NOAF. The inclusion of detailed baseline characteristics, procedural data, and long-term follow-up provided a robust dataset for identifying predictors of NOAF and assessing its impact on clinical outcomes. In addition, the use of multivariate logistic regression analysis enabled the identification of independent risk factors, offering valuable insights for risk stratification and patient management.

Another strength lies in the study's focus on a specific patient population—those with severe aortic stenosis undergoing TAVR—which is particularly relevant given the increasing use of TAVR in both high-risk and low-risk patients. By elucidating the relationship between NOAF and long-term outcomes in this population, our findings have important implications for clinical practice, particularly in terms of optimizing post-procedural care and implementing preventive strategies for NOAF.

The findings of this study have important clinical implications for the management of TAVR patients. The identification of risk factors for NOAF, such as new-onset RBBB, diabetes, and severe tricuspid regurgitation, can help clinicians identify high-risk patients who may benefit from closer monitoring and targeted interventions. In addition, the association between NOAF and increased hospital readmissions highlights the need for optimized post-procedural care, including aggressive management of arrhythmias and comorbid conditions.

Future research should focus on developing and evaluating strategies to prevent NOAF in TAVR patients, such as the use of prophylactic antiarrhythmic medications or minimally invasive techniques to reduce atrial strain during the procedure. In addition, studies should explore the long-term impact of NOAF on quality of life, functional status, and healthcare costs, as these outcomes are critical for understanding the full burden of NOAF in this population.

The limitations of the present study should be acknowledged: (1) The retrospective, single-center nature of the analysis, which may limit the generalizability of the findings. (2) The potential for unmeasured confounding factors or missing data that could influence the observed associations. (3) The reliance on electrocardiographic data, which may not fully capture the complexity of cardiac conduction disturbances in this population. (4) The need for longer-term follow-up to assess the durability of the observed event rates and to identify any emerging trends over a more extended period.

## 5. Conclusions

In conclusion, this study provides valuable insights into the incidence, risk factors, and prognostic implications of NOAF following TAVR. While NOAF was not associated with increased mortality in this cohort, it significantly contributed to higher rates of hospital readmissions and recurrent cardiovascular events. These findings underscore the importance of close monitoring and proactive management of NOAF in TAVR patients, with the ultimate goal of improving long-term outcomes and reducing healthcare utilization.

## Figures and Tables

**Table 1:** Baseline and procedural characteristics (*n* = 601).

Factor	SR/SR group (*n* = 545)	SR/AF group (*n* = 56)	*p*
ECG before procedure			
Heart rate	71 (63, 81)	68.5 (63.3, 80)	0.574
P width	117 (102.5, 122)	110 (100, 120)	0.026
PR interval	176 (159, 195.5)	173 (151.3, 203.5)	0.500
First-degree AVB	95 (17.4)	15 (26.8)	0.085
QRS width	96 (88, 108)	97 (89.3, 108.8)	0.534
LBBB	22 (4)	2 (3.6)	1.000^∗^
RBBB	36 (6.6)	4 (7.1)	1.000
RBBB and LAFB/LAPB	10 (1.8)	2 (3.6)	0.702
ECG after procedure			
New first-degree AVB	111 (20.4)	3 (5.4)	0.006
New second-degree AVB type 1	7 (1.3)	0 (0.0)	1.000^∗^
New high-degree AVB	66 (12.1)	11 (19.6)	0.108
QRS width	111.50 (94, 149.8)	105 (94, 153.3)	0.736
QRS change	5.5 (−1, 43)	4 (−2, 31)	0.467
New RBBB	35 (6.4)	8 (14.3)	0.057
New LBBB	175 (32.1)	20 (35.7)	0.583
New RBBB and LAFB/LAPB	23 (4.2)	5 (8.9)	0.208
Baseline			
Age, years	75 (70, 80)	75.5 (71.3, 80.8)	0.297
Body mass index, kg/m^2^	22.9 (20.3, 25.1)	23.3 (21.2, 24.8)	0.502
Body surface area, m^2^	1.6 (1.5, 1.7)	1.7 (1.5, 1.8)	0.564
STS score,%	4.2 (2.5, 7.3)	4.9 (3.0, 8.2)	0.157
Male	311 (57.1)	32 (57.1)	0.991
Valve type			0.084^∗^
BAV type 0	140 (25.7)	15 (26.8)	
BAV type 1	139 (25.5)	8 (14.3)	
BAV type 2	5 (0.9)	2 (3.6)	
TAV	261 (47.9)	31 (55.4)	
Tobacco smoker	102 (18.7)	8 (14.3)	0.414
Hypercholesterolemia	95 (17.4)	17 (30.4)	0.018
Hypertension	293 (53.8)	27 (48.2)	0.428
Diabetes mellitus	110 (20.2)	17 (30.4)	0.076
Prior angina	174 (31.9)	12 (21.4)	0.106
Prior syncope	44 (8.1)	4 (7.1)	1.000
NYHA functional class			0.497^∗^
I	3 (0.6)	1 (1.8)	
II	107 (19.6)	11 (19.6)	
III	274 (50.3)	22 (39.3)	
IV	161 (29.5)	22 (39.3)	
Pure AS	336 (61.7)	38 (67.9)	0.362
Prior MI	10 (1.8)	1 (1.8)	1.000
Prior PCI	69 (12.7)	4 (7.1)	0.229
Prior CPR	1 (0.2)	0 (0)	1.000^∗^
Prior CABG	2 (0.4)	0 (0)	1.000^∗^
Prior stroke	22 (4)	5 (8.9)	0.179
Peripheral vascular disease	67 (12.3)	10 (17.9)	0.236
Prior surgical replacement of a valve	1 (0.2)	0 (0)	1.000^∗^
Prior pacemaker	5 (0.9)	0 (0)	1.000^∗^
COPD	104 (19.1)	14 (25)	0.288
Other type of lung disease	71 (13)	6 (10.7)	0.622
Malignancy	24 (4.4)	3 (5.4)	1.000
Pulmonary hypertension	41 (7.5)	4 (7.1)	1.000
Dialysis	6 (1.1)	0 (0)	1.000^∗^
Gastrointestinal bleeding	16 (2.9)	1 (1.8)	0.943
Laboratory examinations			
Leukocyte count (10^9^/L)	6.1 (5, 7.4)	6.8 (5.6, 7.9)	0.021
Hemoglobin (g/L)	125 (112, 137)	122 (110.5, 136)	0.788
Platelet count (10^9^/L)	163 (133, 205)	174.5 (135.5, 199.8)	0.828
Prothrombin time (s)	13.2 (12.8, 13.8)	13.4 (12.9, 14.1)	0.198
Activated partial prothrombin time (s)	36.6 (34.2, 39.5)	36.2 (33.5, 40.4)	0.918
D-Dimer (μg/L)	490 (320, 940)	455 (320, 1130)	0.859
proBNP (pg/mL)	1835 (535, 5170.5)	1759 (516.1, 6912)	0.881
Creatinine (μmol/L)	75 (61, 97)	75.5 (62.9, 89.8)	0.827
Glomerular filtration rate (mL/min)	59.8 (44.2, 75.1)	58.3 (45.3, 74.9)	0.890
Total bilirubin (μmol/L)	12.2 (9.7, 16.2)	11.1 (8.9, 15.6)	0.178
Indirect bilirubin (μmol/L)	9.8 (7.8, 12.9)	8.8 (7.4, 12.2)	0.239
Albumin (g/L)	37.3 (34.9, 39.5)	37.6 (34.9, 39.4)	0.599
Alanine aminotransferase (U/L)	17 (12, 25)	15 (12, 22)	0.483
Aspartate aminotransferase (U/L)	24 (20, 30)	23 (19.3, 33)	0.744
Creatine kinase isoenzyme (U/L)	12 (9, 15.5)	12 (9, 14)	0.839
Troponin T (ng/mL)	0.02 (0.01, 0.05)	0.02 (0.01, 0.04)	0.889
Echocardiography			
LVEDd (cm)	4.8 (4.3, 5.4)	5.1 (4.3, 5.6)	0.157
LA (cm)	4.1 (3.7, 4.5)	4.2 (3.8, 4.7)	0.057
LVEF (%)	60.0 (50.4, 64.7)	58.9 (40.3, 66.3)	0.464
Maximum velocity (m/s)	4.8 (4.3, 5.4)	4.5 (4.2, 5.3)	0.123
Mean gradient (mmHg)	53.0 (42.0, 68.0)	47.5 (41.0, 64.3)	0.147
AVA (cm^2^)	0.6 (0.5, 0.8)	0.6 (0.5, 0.8)	0.942
MS (0–3)			0.798^∗^
No	520 (95.4)	53 (94.6)	
Mild	20 (3.7)	2 (3.6)	
Moderate	5 (0.9)	1 (1.8)	
Grade of aortic regurgitation			0.547^∗^
No	90 (16.5)	11 (19.6)	
Mild	227 (41.7)	23 (41.1)	
Moderate	161 (29.5)	16 (28.6)	
Severe	61 (11.2)	6 (10.7)	
Mild-to-moderate	5 (0.9)	0 (0)	
Moderate-to-severe	1 (0.2)	0 (0)	
MR			0.333^∗^
No	117 (21.5)	5 (8.9)	
Mild	311 (57.1)	38 (67.9)	
Moderate	93 (17.1)	11 (19.6)	
Severe	14 (2.6)	2 (3.6)	
Mild-to-moderate	9 (1.7)	0 (0)	
Moderate-to-severe	1 (0.2)	0 (0)	
TR			0.016
No	223 (40.9)	16 (28.6)	
Mild	271 (49.7)	32 (57.1)	
Moderate	44 (8.1)	4 (7.1)	
Severe	7 (1.3)	4 (7.1)	
CT examination			
Maximum diameter (mm)	27.2 (25.4, 29.2)	27.5 (25.7, 30.1)	0.317
Minimum diameter (mm)	21.3 (19.7, 22.9)	21.7 (19.5, 22.9)	0.980
Average diameter (mm)	24.3 (22.7, 26)	24.8 (23, 25.8)	0.513
Area (mm^2^)	452.1 (399, 522.0)	465.3 (413.9, 507.5)	0.532
Area-derived diameter (mm)	24 (22.6, 25.8)	24.3 (23, 25.4)	0.571
Perimeter (mm)	76.8 (72.1, 82.5)	78.5 (73.6, 82)	0.351
Perimeter-derived diameter (mm)	24.5 (23, 26.3)	25 (23.4, 26.1)	0.408
STJ diameter (average) (mm)	30.3 (27.4, 33)	30.5 (28.6, 33.4)	0.321
STJ height (mm)	21.4 (19.1, 24)	21.8 (19.5, 24.6)	0.269
Ascending-aorta diameter (4 cm) (mm)	37.2 (34.3, 40.5)	37.2 (35.4, 40.8)	0.717
Ascending-aorta diameter (maximum) (mm)	40.3 (36.4, 44.4)	39.3 (37.2, 43.9)	0.766
RCA height (mm)	16.3 (14.4, 18.5)	16.7 (14.8, 18.6)	0.662
LM height (mm)	14.2 (12.1, 16.7)	14.1 (11.6, 17.8)	0.930
Aortic root angle (°)	51 (45, 58)	52 (47, 57)	0.796
Calcified	526 (97.2)	54 (98.2)	1.000
Calcification grade (0–4)			1.000
No	14 (2.6)	1 (1.8)	
Mild	77 (14.2)	10 (18.2)	
Moderate	180 (33.3)	14 (25.5)	
Severe	194 (35.9)	24 (43.6)	
Extremely severe	76 (14.0)	6 (10.9)	
Calcification distribution	362 (66.9)	30 (54.5)	0.066
Procedure			
Sheath size (mm)	20 (18, 20)	18 (18, 20)	< 0.001
Anesthesia type			0.026^∗^
Local	485 (89.0)	43 (76.8)	
General	52 (9.5)	11 (19.6)	
Switch to general anesthesia	8 (1.5)	2 (3.6)	
Access			0.439^∗^
Femoral artery	532 (97.6)	54 (96.4)	
Subclavian artery	1 (0.2)	0 (0)	
Carotid artery	12 (2.2)	2 (3.6)	
Predilatation	523 (96.0)	52 (92.9)	0.457
Valve type			0.253^∗^
Self-expansion	483 (88.6)	51 (91.1)	
Balloon dilation	46 (8.4)	2 (3.6)	
Mechanical expansion	16 (2.9)	3 (5.4)	
Valve size	26 (25, 29)	26 (26, 29)	0.065
Post-dilatation	312 (57.2)	31 (55.4)	0.785
Complications			
Blood transfusion	33 (6.1)	0 (0)	0.113
Hydropericardium	47 (8.6)	13 (23.2)	< 0.001
AR primarily paravalvular			0.828
No	235 (43.1)	20 (35.7)	
Mild	274 (50.3)	34 (60.7)	
Moderate	32 (5.9)	2 (3.6)	
Mild-to-moderate	4 (0.7)	0 (0)	
Vascular complications	34 (6.2)	1 (1.8)	0.291
Annular rupture	1 (0.2)	0 (0)	1.000^∗^
Coronary obstruction	4 (0.7)	0 (0)	1.000^∗^
Circulation collapse	14 (2.6)	0 (0)	0.454

*Note:* LM: left main coronary artery; LAPB: left posterior fascicular block.

Abbreviations: AR, aortic regurgitation; AS, aortic stenosis; AVA, aortic valve area; AVB, atrioventricular block; BAV, bicuspid aortic valve; CABG, coronary artery bypass graft; COPD, chronic obstructive pulmonary disease; CPR, cardiopulmonary resuscitation; CT, computed tomography; ECG, electrocardiogram; LA, left atrium; LAFB, left anterior fascicular block; LBBB, left bundle branch block; LVEDd, left ventricular end-diastolic diameter; LVEF, left ventricular ejection fraction; MI, myocardial infarction; MR, mitral regurgitation; MS, mitral stenosis; PCI, percutaneous coronary intervention; proBNP, pro-B-type natriuretic peptide; RBBB, right bundle branch block; RCA, right coronary artery; STJ, sinotubular junction; STS, Society of Thoracic Surgeons; TAV, tricuspid aortic valve; TR, tricuspid regurgitation.

^∗^Fisher's exact test.

**Table 2:** Univariable and multivariable analyses of risk factors for atrial fibrillation (AF).

Variable	Univariable analysis (OR, 95% CI; *p* value)	Multivariable analysis (adjusted OR, 95% CI; *p* value)
RBBB	2.45 (1.08–5.56; *p*=0.032)	3.631 (1.519–8.681; *p*=0.004)
Diabetes mellitus	1.73 (0.94–3.18; *p*=0.076)	2.104 (1.090–4.063; *p*=0.027)
Hydropericardium	3.21 (1.60–6.45; *p*=0.001)	3.246 (1.527–6.901; *p*=0.002)
Severe TR	5.78 (1.62–20.6; *p*=0.007)	10.322 (2.535–42.110; *p*=0.001)
Hypercholesterolemia	2.06 (1.13–3.76; *p*=0.018)	1.926 (0.993–3.734; *p*=0.052)
First-degree AV block	1.74 (0.92–3.29; *p*=0.085)	1.68 (0.88–3.21; *p*=0.116)

Abbreviations: CI, confidence interval; OR, odds ratio; RBBB, right bundle branch block; TR, tricuspid regurgitation.

**Table 3:** Impact of NOAF on clinical outcomes: Univariable and multivariable analyses.

Outcome	Time point	SR/SR group (*n* = 545)	SR/AF group (*n* = 56)	Univariable analysis (OR, 95% CI; *p* value)	Multivariable analysis (adjusted OR, 95% CI; *p* value)
All-cause mortality	1 year	29 (5.3%)	1 (1.8%)	0.33 (0.04–2.45; *p*=0.240)	0.35 (0.05–2.56; *p*=0.324)
	3 years	52 (9.5%)	4 (6.1%)	0.62 (0.22–1.78; *p*=0.412)	0.65 (0.23–1.85; *p*=0.356)
	5 years	73 (13.4%)	7 (12.5%)	0.92 (0.41–2.08; *p*=0.571)	0.95 (0.42–2.15; *p*=0.498)

Cardiovascular death	1 year	11 (2.0%)	0 (0%)	N/A	N/A
	3 years	21 (3.9%)	0 (0%)	N/A	N/A
	5 years	29 (5.4%)	0 (0%)	N/A	N/A

Hospital readmissions	1 year	125 (22.9%)	18 (32.1%)	1.61 (0.89–2.91; *p*=0.123)	1.68 (0.92–3.08; *p*=0.091)
	3 years	180 (33.0%)	27 (48.2%)	1.89 (1.12–3.18; *p*=0.023)	1.95 (1.15–3.31; *p*=0.013)
	5 years	206 (37.8%)	30 (53.6%)	1.91 (1.13–3.23; *p*=0.021)	1.98 (1.17–3.35; *p*=0.011)

Myocardial infarction	1 year	2 (0.4%)	1 (1.8%)	4.78 (0.43–53.2; *p*=0.255)	4.82 (0.44–53.5; *p*=0.248)
	3 years	4 (0.7%)	1 (1.8%)	2.56 (0.28–23.4; *p*=0.388)	2.58 (0.29–23.6; *p*=0.382)
	5 years	4 (0.7%)	1 (1.8%)	2.56 (0.28–23.4; *p*=0.388)	2.58 (0.29–23.6; *p*=0.382)

Stroke	1 year	11 (2.0%)	0 (0%)	N/A	N/A
	3 years	17 (3.1%)	2 (3.6%)	1.16 (0.26–5.18; *p*=0.611)	1.18 (0.27–5.23; *p*=0.245)
	5 years	19 (3.5%)	2 (3.6%)	1.03 (0.23–4.56; *p*=0.611)	1.05 (0.24–4.62; *p*=0.245)

Disabling stroke	1 year	9 (1.7%)	0 (0%)	N/A	N/A
	3 years	11 (2.0%)	0 (0%)	N/A	N/A
	5 years	11 (2.0%)	0 (0%)	N/A	N/A

Bleeding events	1 year	16 (4.8%)	3 (5.4%)	1.13 (0.32–4.02; *p*=0.611)	1.15 (0.33–4.05; *p*=0.214)
	3 years	36 (6.6%)	3 (5.4%)	0.81 (0.24–2.71; *p*=0.939)	0.83 (0.25–2.75; *p*=0.214)
	5 years	42 (7.7%)	5 (8.9%)	1.17 (0.45–3.05; *p*=0.950)	1.19 (0.46–3.08; *p*=0.214)

Fatal bleeding	1 year	3 (0.6%)	2 (3.6%)	6.34 (1.03–39.1; *p*=0.071)	6.45 (1.05–39.8; *p*=0.071)
	3 years	6 (1.1%)	2 (3.6%)	3.34 (0.66–16.9; *p*=0.166)	3.38 (0.67–17.1; *p*=0.166)
	5 years	8 (1.5%)	2 (3.6%)	2.45 (0.51–11.8; *p*=0.237)	2.48 (0.52–11.9; *p*=0.237)

Acute kidney failure	1 year	1 (0.2%)	1 (1.8%)	9.56 (0.59–155; *p*=0.178)	9.62 (0.60–156; *p*=0.178)
	3 years	4 (0.7%)	1 (1.8%)	2.56 (0.28–23.4; *p*=0.388)	2.58 (0.29–23.6; *p*=0.382)
	5 years	5 (0.9%)	1 (1.8%)	2.03 (0.23–17.7; *p*=0.445)	2.05 (0.24–17.9; *p*=0.445)

## Data Availability

The data that support the findings of this study are available from the corresponding author upon reasonable request.
